# The spectrum of HIV mother-to-child transmission risk

**DOI:** 10.7448/IAS.17.4.19703

**Published:** 2014-11-02

**Authors:** Veronique Reliquet, Norbert Winer, Natacha Chereau, Elise Launay, Aurore Lamberet, Elisabeth Andre-Garnier, Francois Raffi, Cecile Brunet

**Affiliations:** 1 Infectious Diseases, CHU Nantes, Nantes, France; 2 Obstetrics and Perinatal Medicine, CHU Nantes, Nantes, France; 3 Pediatrics, Hôpital Mère-Enfant, CHU Nantes, Nantes, France; 4 CHU Nantes, Virology Laboratory, Nantes, France

## Abstract

**Introduction:**

With the implementation of combined antiretroviral therapy (cART) and prevention of mother-to-child transmission (MTCT) we observed dramatic decreases in rates of perinatal MTCT of HIV, 0.3% in France in women with plasma viral load (pVL) <50 c/mL at delivery. We describe a case of MTCT which occurred despite virologic suppression of the mother at delivery, the first case in our centre since 2002.

**Description of the case:**

A 26-year-old black woman, Guinea-native, living in France since 2007, was diagnosed with HIV-1 CRF02 in 2008 and lost to follow-up since November 2012 after second delivery (2 female born in March 2009 and October 2012, uninfected). Third pregnancy began in July 2013 and baseline characteristics in September were as follows: week 13 of gestational age (GA), CDC stage A, CD4 317/mm^3^, pVL 4.89 log c/mL. cART with abacavir/lamivudine and atazanavir/ritonavir 300/100 mg daily (qd) was introduced. VL decreased to 2.4 log c/mL in 4 weeks and CD4 increased to 456/mm^3^. In December, at week 22 of GA, viral rebound at 4.14 log c/mL due to sub-optimal maternal adherence was observed. After counselling, pVL decreased to 1.69 log c/mL in March 2014, at week 35 of GA and 1.3 log c/mL at delivery. As pVL was <400 c/mL at week 36 of GA, vaginal delivery with IV zidovudine was decided. However, because of poor/uncertain maternal adherence to cART, the neonate was treated with a combination of 2 drugs (lamivudine-nevirapine) with the 4-week zidovudine regimen, until the result of delivery pVL. This combination was stopped at day 2 when maternal delivery pVL (22 c/mL) was received and standard oral zidovudine prophylaxis was continued. Infant was tested for HIV infection at baseline (day 3) and found to be HIV-infected (HIV-RNA 60 c/mL) attesting in-utero HIV transmission. On day 15, zidovudine prophylaxis was discontinued and treatment for HIV infection initiated with standard cART according to the French Paediatric Antiretroviral Guidelines.

**Conclusions:**

The risk of HIV acquisition is low in infants born to women who receive standard cART during pregnancy and labour and achieve undetectable VL at delivery. However, transmission remains a hazard, with possibility of in-utero infection during episodes of non-adherence, and the risk of a possible MTCT has to be mentioned to all pregnant women.

